# Adverse events during short- and long-term exposure to low-dose weekly methotrexate for rheumatic diseases

**DOI:** 10.1093/rap/rkad038

**Published:** 2023-04-05

**Authors:** Mark Garton, Yee-Ping Teoh, David Watson

**Affiliations:** Department of Rheumatology, Wrexham Maelor Hospital, Wrexham, UK; Department of Chemical Pathology, Wrexham Maelor Hospital, Wrexham, UK; Department of Haematology, Wrexham Maelor Hospital, Wrexham, UK

Key message

- Acute and chronic toxicity during low-dose methotrexate is common and deserves improved recognition and mitigation.

Dear Editor, Sherbini *et al.* [1] recently reported the scope and scale of MTX-associated toxicity during the first year of treatment for rheumatoid arthritis and emphasize the challenge of communicating prospectively these risks to patients. Almost four-fifths of their cohort admitted adverse events (AEs) and nearly one-third reported nausea, fatigue or neurological symptoms. Their structured questionnaire may have inflated reporting of short-term predefined AEs, but hazards of chronic exposure (e.g. malignancy) were not assessed.

We audited electronic case records (available from 2007 onwards) of all adults scheduled for review between February and April 2018 in our outpatient rheumatology clinics (serving a catchment population of nearly 300 000). This project was registered with the local Betsi Cadwaladr University Health Board audit department as a service evaluation (study 202, 2020); ethical approval and informed consent were therefore not required. Of 1448 attenders, we identified 758 (495 female) patients currently or previously prescribed MTX for a mean of 5.8 years (range 1 week–23.8 years), mostly for rheumatoid (70%) or psoriatic (18%) arthritis. The mean age at starting treatment was 55.8 (s.d. 14.8 years) and the median peak dosage was 20 mg/week. MTX duration was known (83.5%), estimated (4.5%) or censored from the earliest confirmed exposure (8.3%) and was unknown for 28 (3.7%) patients. In line with national guidelines, all patients prescribed MTX in our unit were also prescribed a folic acid supplement, at a minimal dose of 5 mg once weekly, taken 2 days after the MTX.

We documented the type and timing of AEs considered MTX-related by attending clinicians and incident cancers diagnosed during/after MTX exposure. During 4175 years of follow-up, 451 (59.5%) patients experienced one or more AEs after a median of 1435 days (range 1–7745) among 430 patients with available data. Various AEs were recorded (Table 1), most often gastrointestinal, prompting permanent withdrawal in 291 (38.4%) subjects, with 1 in 6 stopping treatment within 12 months. Infections (mainly chest, skin and urinary tract, but also herpes/other DNA viruses) and serum transaminitis were also commonly reported. Although hair loss and neuropsychological symptoms accounted for <10% of AEs, more than two-thirds of these patients withdrew MTX permanently. A total of 35 patients developed 42 malignancies (half of keratinocyte origin) during treatment in all but 5 subjects, with none confirmed within the first year of treatment. We separately identified 33 patients with interstitial lung disease (ILD), including 10 with possible MTX-induced pneumonitis, but incomplete records and/or competing diagnoses often precluded distinction from spontaneous deteriorations in pre-existing ILD.

**Table 1.** Frequency and timing of reported AEs among 758 patients receiving weekly low-dose MTX for rheumatic diseases

Domain	Patients with an AE, *n* (%)	Patients withdrawing MTX, *n* (%)	Exposure, days, median (range), (*n* with data)
Gastrointestinal^a^	161 (21.2)	101 (62.7)	235 (1–5890), (*n* *=* 121)
Hepatic (peak ALT ≥ ULN)^b^	122 (16.1)	79 (64.8)	499 (27–4288), (*n* = 95)
Infection^c^	113 (14.9)	70 (61.9)	1130 (28–6091), (*n* = 97)
Haematological^d^	64 (8.4)	38 (59.4)	1388 (96–5512), (*n* = 48)
Neuropsychological^e^	45 (5.9)	32 (71.1)	421 (23–5265), (*n* = 32)
Oncological^f^	35 (4.6)	20 (57.1)	1827 (709–5465), (*n* = 30)
Cutaneous			
Alopecia	28 (3.7)	21 (75)	336 (54–3897), (*n* = 16)
Nodules	8 (1.1)	4 (50)	1294 (1036–1931), (*n* = 5)
Allergy	6 (0.8)	6 (100)	38 (7–447), (*n* = 4)

ALT: alanine transaminase; ULN: upper limit of normal.

a Nausea, ulcers, diarrhoea or vomiting.

b Peak serum ALT (18 ≤ 2 × ULN, 87 > 2 × ULN, 17 missing).

c Infections of chest, skin, urine, bone, ENT and viral (including 4 herpes zoster, 2 HSV and 1 each of EBV, HPV and orf).

d Leucopenia, neutropenia and thrombocytopenia.

e Depression, fatigue, headache, cognitive impairment and dizziness.

f Skin (8 basal cell carcinoma, 5 squamous cell carcinoma, 1 malignant melanoma, 2 pre-malignant), lymphocytic (4 lymphoma, 2 leukaemia), colorectal (6), breast (4), lung (3), prostate (2), vulvovaginal (2) and single cases of acoustic neuroma, nasopharyngeal and metastases with unknown primary.

Like Sherbini *et al*., we found no significant difference in the mean weekly MTX dosage among those with [19.5 mg (s.d. 4.7)] or without [19.6 mg (s.d. 4.5)] AEs, although the former group subsequently decreased their mean weekly dosage from 18.3 to 15.7 mg (paired *t*-test, *P* < 0.001). We additionally demonstrated a trend to variable latencies for different AE domains, with median MTX exposures being shorter for allergy, gastrointestinal disturbance and alopecia and longer for blood dyscrasias and malignancies compared with transaminitis or neuropsychological symptoms.

Our large single-centre observational study has some limitations. Opportunistic AE reporting and clinic-based patient selection risk underestimating drug toxicity due to ascertainment and survivorship bias, respectively, while the absence of suitable controls restricts causal inference. Nonetheless, our real-life results may still provide useful insights into the spectrum and timing of MTX-associated AEs during short- and long-term use. Also, our results are consistent with the randomized controlled Cardiovascular Inflammation Reduction Trial (CIRT) [2] of low-dose MTX for secondary prevention of cardiovascular disease. The CIRT reported higher rates of gastrointestinal, pulmonary, infectious and haematologic AEs among MTX-treated patients versus placebo during a mean follow-up of 2.3 years, and higher all-cancer incidence [hazard ratio (HR) 1.72], driven largely by an excess of non-basal cell skin cancers (HR 3.08). Intriguingly, survival curves for malignancy (and infection) in the CIRT only separated from placebo 12–18 months after starting MTX, so chronic exposure might promote a broader range of malignancies, as suggested by large observational studies [3, 4]. Low-dose MTX may be more immunosuppressive than is commonly believed and we speculate that reactivation of oncogenic DNA viruses (such as EBV and HPV) during therapy, and perhaps impaired DNA repair, might be aetiologically relevant in some patients [5].

Neurological AEs such as fatigue, depression and headache are easily overlooked, but are emerging as important determinants of mental health quality of life during MTX therapy [6], and our data suggest these side effects and alopecia may particularly predispose to treatment withdrawal. Notably, we found no overall gender differences in reporting of individual AEs, except for alopecia, which was almost exclusively reported by female patients.

As MTX remains an effective core drug for controlling inflammatory rheumatic disease, strategies to predict and control AEs are clearly important. Dosage per se may not reliably predict toxicity, but lower initial doses for all might reduce AEs for some without losing efficacy [7]. This is particularly important for those with traditional risk factors (e.g. increasing age, reduced eGFR, hypoalbuminemia), but other host-specific characteristics such as prior bone marrow transplant might also justify caution. Reactivation of latent viral infections may cause direct host damage or contribute indirectly to downstream oncogenesis; either might be mitigated by selective immunization, chemoprevention or specific antiviral therapies. Above all, we must focus on those AEs that are serious and/or most intrusive, as these may adversely impact on long-term adherence and persistence with treatment.

Patients need and deserve high-quality information regarding inevitable trade-offs between benefits and risks of treatment [8], as well as alternative strategies, to support truly informed consent and shared decision making. While potential treatment benefits are often perceived as more important than even serious hazards, patient preferences are variable, underlining the need for patient-centred choice [9]. Future research should prioritize AEs associated with chronic exposure, which will require careful long-term follow-up of treated patients.

## Data Availability

The data underlying this article cannot be shared publicly for the privacy of individuals who participated in the study.
